# The coevolution of cognition and sociality

**DOI:** 10.1098/rstb.2024.0110

**Published:** 2025-06-26

**Authors:** Luca G. Hahn, Andoni S. E. Sergiou, Josh J. Arbon, Ines Fürtbauer, Andrew J. King, Alex Thornton

**Affiliations:** ^1^ Centre for Ecology and Conservation, University of Exeter, Penryn TR10 9FE, UK; ^2^ Department of Biosciences, Swansea University, Swansea SA2 8PP, UK; ^3^ School of Biological Sciences, University of Bristol, Bristol BS8 1TQ, UK

**Keywords:** cognition, coevolutionary feedback loop, information, sociality, socio-ecological trade-off, uncertainty

## Abstract

Cognition serves to resolve uncertainty. Living in social groups is widely seen as a source of uncertainty driving cognitive evolution, but sociality can also mitigate sources of uncertainty, reducing the need for cognition. Moreover, social systems are not simply external selection pressures but rather arise from the decisions individuals make regarding who to interact with and how to behave. Thus, an understanding of *how* and *why* cognition evolves requires careful consideration of the coevolutionary feedback loop between cognition and sociality. Here, we adopt ideas from information theory to evaluate how potential sources of uncertainty differ across species and social systems. Whereas cognitive research often focuses on identifying human-like abilities in other animals, we instead emphasize that animals need to make adaptive decisions to navigate socio-ecological trade-offs. These decisions can be viewed as feedback loops between perceiving and acting on information, which shape individuals’ immediate social interactions and scale up to generate the structure of societies. Emerging group-level characteristics such as social structure, communication networks and culture in turn produce the context in which decisions are made and so shape selection on the underlying cognitive processes. Thus, *minds shape societies and societies shape minds*.

This article is part of the Theo Murphy meeting issue ‘Selection shapes diverse animal minds’.

## 1. Introduction

Cognition serves to predict and control the outcomes of an individual’s interactions with the environment—in other words, to reduce uncertainty, by collecting, processing, storing and acting upon information [1]. Uncertainty refers to the precision with which a prediction can be made given available information [2,3]. To understand how selection shapes cognition, it is necessary to establish the sources of uncertainty in the environment.

The social environment is widely seen as a key source of uncertainty, promoting the evolution of neural architectures and cognitive traits that enable individuals to track information about social interactions and reduce uncertainty to optimize social decisions. This view, known broadly as the *Social Intelligence Hypothesis* [4–8], has been highly influential but remains contentious [9,10] for several reasons. First, existing evidence is rather mixed. For instance, reports of positive associations between aspects of the social environment (e.g. group size or monogamous relationships) and measures of neuroanatomy or cognitive performance are generally correlational [11], with numerous studies reporting contradictory findings (e.g. [12,13]). Second, while the social environment may generate uncertainty [2], it can also reduce it—for example by enabling individuals to use information generated by others [14,15]. Finally, viewing the social environment as an external pressure driving the evolution of cognitive processes neglects the fact that social structure is itself the outcome of many individuals’ decisions regarding who to interact with and how to behave [16]. In other words, *minds shape societies and societies shape minds*. A more nuanced understanding of *how* and *why* cognition evolves to navigate the socio-ecological environment requires consideration of the specific circumstances in which social life generates uncertainty, and the coevolutionary feedback between cognition (i.e. information-gathering and decision-making [1]) and sociality (i.e. social interactions, relationships and structure [17]).

**Figure 1.**
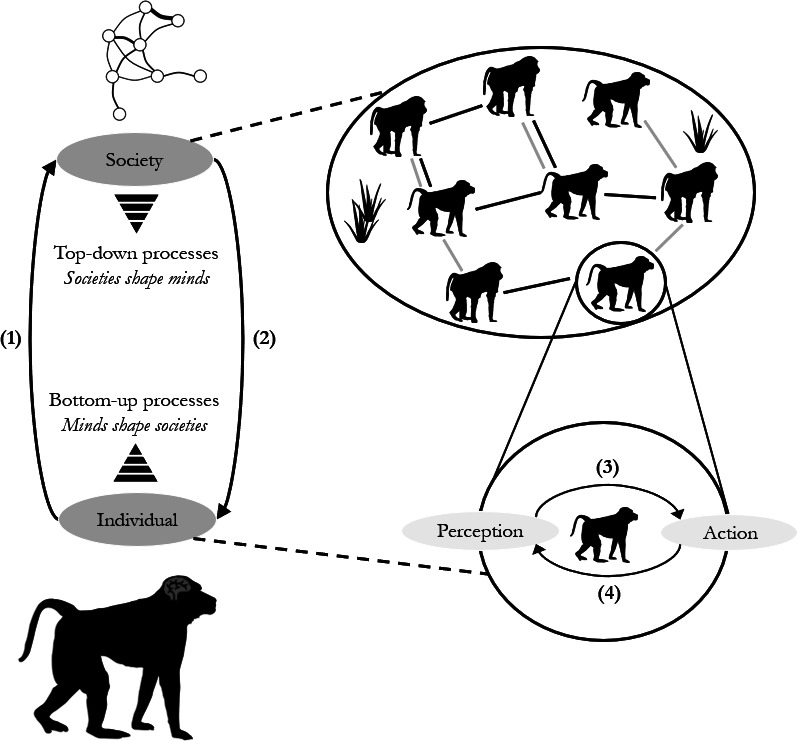
Coevolutionary feedback loop between cognition and sociality. Minds shape societies via bottom-up processes (arrow 1), and societies shape minds via top-down processes (arrow 2). Societies can be conceptualized as social networks of interactions between individuals in different contexts, such as grooming or mating (symbolized by lines between individuals, with colours representing different social contexts). The social network provides the context for and is shaped by individual decision-making. Individual decision-making unfolds through a perception–action feedback loop (arrows 3 and 4; see §4) by which individuals use and update their knowledge to increase their payoffs. Individual decisions and group outcomes (e.g. social structure) are embedded in and influenced by external ecological factors, such as resource availability and distribution. Animal icons from *PhyloPic* (Fabio Machado and Kai Caspar).

Uncertainty in the social environment and coevolutionary feedback between sociality and cognition have been discussed in previous works. For instance, Taborsky & Oliveira argue that an individual’s ability to use social information to optimize social decisions, termed ‘social competence’, impacts fitness [18]. More socially competent individuals may achieve better outcomes from social interactions, leading them to engage more optimally and frequently with others, which could then drive further improvements in social competence [19]. Wascher *et al*. emphasize that cognitive abilities, such as individual recognition, learning and memory, are required for managing social relationships, and these abilities can influence how individuals benefit from forming and maintaining social relationships [20]. Other authors, for example Kulahci *et al*., focus on feedback loops in the context of social learning: social relationships influence information transmission, but the acquisition of knowledge can modify the value of individuals as social partners, thereby shaping their social relationships [21–23]. Cantor *et al*. present a broader review of the ecological and evolutionary consequences of such individual-to-society feedback loops [16], but without a specific focus on cognition. In individual-to-society feedback loops, individual decisions scale up to shape the local and global social environment (i.e. bottom-up processes from individuals to groups), which feeds back by shaping individual behaviour (i.e. top-down processes from groups to individuals). A broader synthesis of these different related ideas is currently lacking, and a better understanding of the coevolution of cognition and sociality thus requires a broader integration of bottom-up and top-down processes (figure 1).

In this review, we unify and extend existing approaches to understand how cognition and sociality coevolve, by considering feedback loops at individual and group levels (figure 1). We start by discussing how hypotheses linking sociality and cognitive evolution can benefit from incorporating concepts from information theory [3] to identify the contexts in which selection is likely to favour cognitive abilities to reduce social uncertainty (§2). We then discuss how cognition could allow animals to navigate different uncertainty problems, focusing on socio-ecological trade-offs (§3). Section 4 focuses on the bottom-up consequences of individual decisions, considering how feedback between perception and action [24] enables animals to update their knowledge and fine-tune their social behaviour, and how this in turn shapes their social environment. Finally, we discuss how group-level phenomena, such as social structure, communication networks and culture [25,26], are a consequence of many individual decisions, and how these group-level phenomena can generate selection for socio-cognitive traits. With these bottom-up and top-down influences at play, the coevolutionary feedback loop of sociality and cognition helps shape the diversity of animal minds. Crucially, we argue that this approach requires an explicit focus on (i) identifying social sources of uncertainty and information [27,28] and (ii) socio-ecological trade-offs (e.g. [29]) that may govern social decision-making.

## 2. Uncertainty and information about the socio-ecological world

Uncertainty, as conceptualized in information theory, refers to the precision with which agents can predict outcomes of a variable given available information [3]. For instance, if several potential outcomes are predicted to be equally likely, uncertainty is high, whereas uncertainty is low when one outcome is substantially more likely than others. The influence of the Social Intelligence Hypothesis and associated ideas has, perhaps inadvertently, promoted the assumption that social life *per se* is intrinsically uncertain and the major driver of cognitive evolution [9]. However, the extent to which sociality generates uncertainty that can be detected and reduced through cognition (or otherwise) and the precise mechanisms involved often remain unclear, and uncertainty reduction is not always adaptive. For example, using cognition to reduce uncertainty is only valuable in environments that are neither highly predictable nor unpredictable [30] (along a continuum), and the time and energy associated with maintaining neural architecture [31] and collecting and processing information [32] can make uncertainty reduction costly. Furthermore, the informational challenges and opportunities faced by social individuals can be overcome and realized in different ways that we discuss below.

Many animal cognition researchers and proponents of the Social Intelligence Hypothesis have argued that individuals require cognitive mechanisms to deal with the social environment, often emphasizing human-like mechanisms such as ‘theory of mind’ [33,34]. These cognitive traits and their underlying neural architecture have been assumed to involve substantial energetic costs, imposing constraints on their evolution [31]. In contrast, the field of collective animal behaviour emphasizes how simple stereotyped rules of interaction requiring minimal cognitive investment can generate complex group outcomes [35]. Through such ‘self-organization’, cathedral termites (*Nasutitermes triodiae*) are able to build a mound that resembles Gaudí’s famous Basílica de la Sagrada Família in Barcelona without any one individual having a concept of the overall design [36]. However, individual (i.e. cognitive, e.g. via transitive inference) and distributed (e.g. via self-organization) information processing are not mutually exclusive but rather coexist to varying degrees in natural populations, and so it is important to integrate the two perspectives. For instance, bottlenose dolphins (*Tursiops truncates*) and orcas (*Orcinus orca*) form alliances with specific individuals with whom they coordinate activities and cooperate, likely requiring them to track information about past interactions (e.g. [37]). However, they also use cultural markers of group identity (e.g. signature whistles and dialects), which enables them to guide cooperative interactions in larger groups through a more distributed form of information processing without having to track past observations of conspecifics [38]. The extent to which animals rely on individual cognition or distributed forms of information processing will reflect their socio-ecology.

To evaluate how the socio-ecological environment can pose cognitive challenges, we need to determine potential sources of uncertainty as well as the mechanisms and adaptive value of reducing such uncertainty. In many cases, social behaviour can be mediated by non-cognitive processes (e.g. hormonal mechanisms) or through simple heuristics, like acting submissively towards larger or more brightly coloured individuals. Critical factors driving the need for cognitive solutions include the extent of variability in individual behaviour and the degree to which the interests of interacting individuals are aligned under given ecological conditions. Take the example of predator avoidance. Here, cues and signals from other individuals (e.g. fleeing movements and alarm calls) reduce uncertainty about predator risk, enabling large-scale, anonymous information propagation and helping individuals avoid danger [39]. The risk of death means that individuals benefit from responding to social information regardless of which individual(s) produced the information [40]. However, where distinct responses are needed to optimize interactions with different individuals that vary in their behaviour, this is likely to favour a greater reliance on individual-level social information use and decision-making, especially for animals that maintain differentiated social relationships [41]. For example, in birds, simple, stereotyped rules of interaction between neighbours can give rise to coordinated flocking behaviour, but in species that form long-term pair-bonds the value of responding to different neighbouring flock members may differ. This is seen in jackdaws (*Corvus monedula*), where individuals keep track of their pair-bonded partner (their most valuable relationship), maintaining close spatial proximity even when flying amidst hundreds of other flock members [42]. Whenever the interests of individuals that interact repeatedly and behave differently are not aligned, tracking information could be valuable in reducing uncertainty, which could select for cognitive traits such as individual recognition. For example, in paper wasps (*Polistes fuscatus*), conflicts can arise when several queens nest together in patches of finite resources. Selection has, therefore, favoured the evolution of individual facial recognition in this species but not in a closely related species (*Polistes metricus*) where females nest alone and thus do not face competition [43,44]. Similarly, in groups of Western Australian magpies (*Cracticus tibicen dorsalis*) multiple females breed, rates of extra-group paternity are high, within-group relatedness is low, and group members facultatively contribute to alloparental care [45,46]. The resulting conflicts of interest within groups mean that individuals may benefit from tracking information to maintain individualized social relationships to maximize access to resources and breeding opportunities. In larger groups, such cognitive challenges may be intensified. Indeed, Australian magpies growing up in larger social groups perform better in cognitive tasks, indicating that they may have developed cognitive skills to overcome enhanced cognitive challenges. These challenges may include the need to respond differently to multiple different group members depending on their identities (e.g. when ‘negotiating’ contributions to cooperative breeding), inhibit unproductive responses and learn new responses based on the outcomes of past interactions so as to manage social relationships [47]. In contrast, in pied babblers (*Turdoides bicolor*), group size was unrelated to performance in a cognitive test battery involving associative and reversal learning, and inhibitory control [48]. This may be because pied babblers live in cooperatively breeding groups composed of relatives where only dominants breed and subordinates help, with little opportunity to negotiate access to breeding opportunities and thus relatively low uncertainty to be resolved through cognitive means. Similarly, woodpeckers (Picidae) that live in large, stable and cooperative social groups have smaller brains [49], and stable cooperative groups with high levels of relatedness among individuals are also associated with smaller brain size in mammals [50].

**Figure 2.**
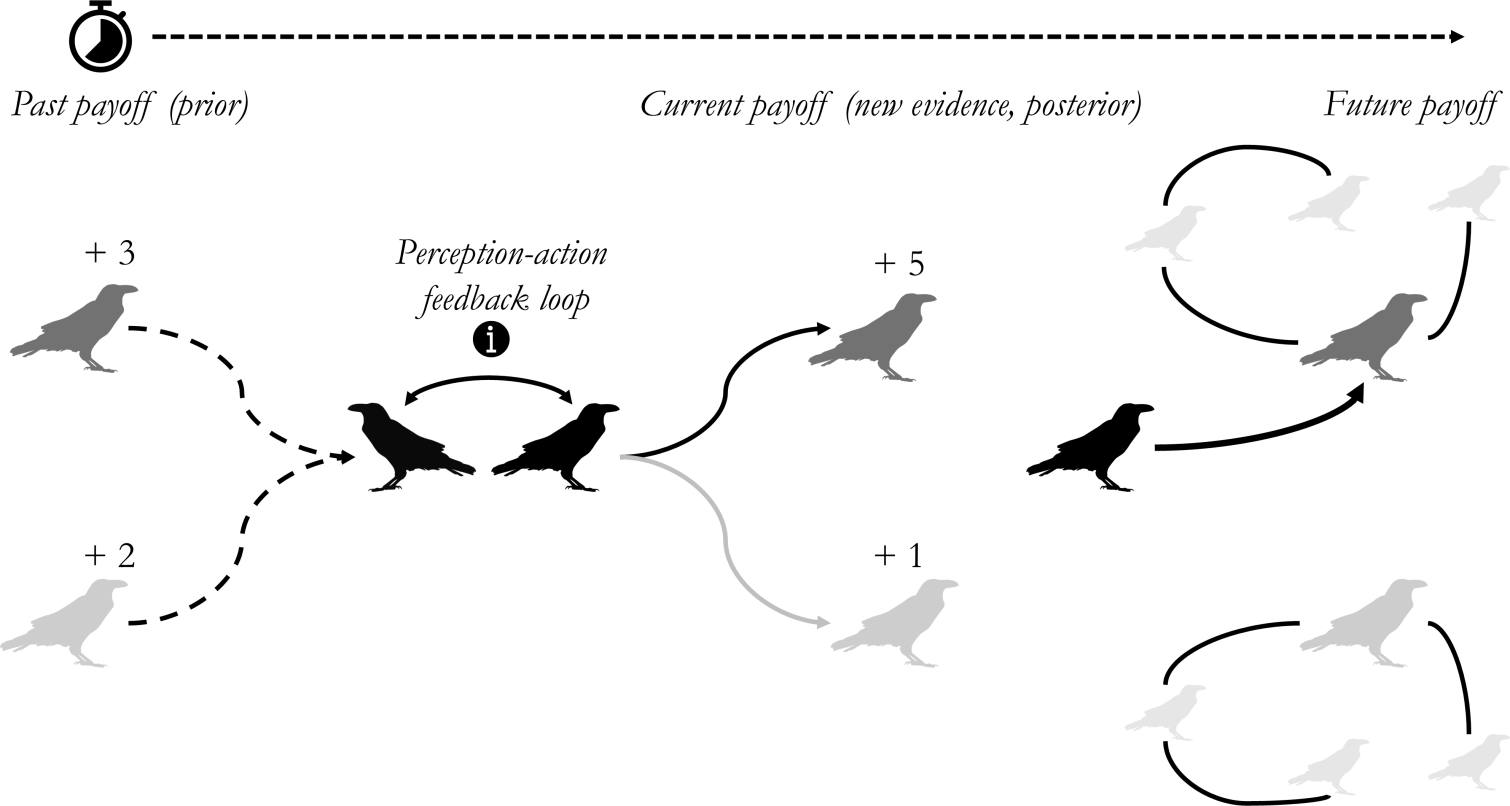
Perception–action feedback loops to overcome socio-ecological trade-offs in biological markets. Here, a focal individual (black) perceives two potential foraging partners and makes subsequent decisions. In this instance, memory of past payoffs (prior) suggest that the two other individuals yield similar payoffs (i.e. there is more uncertainty). New informational input about payoffs, decreasing uncertainty, allows the focal individual to decide about whom to associate with (posterior; bold arrow). This has bottom-up consequences for social structure, which influences whom an individual encounters and who may provide payoffs to that individual in the future. Animal icons from *PhyloPic* (Anthony Caravaggi).

The role of shared interests in modulating social uncertainty varies across the continuum between cooperation and conflict, typically driven by ecological parameters such as resource availability and distribution. Two extremes of this continuum are illustrated by two variants of the Social Intelligence Hypothesis. In situations characterized by relatively high conflicts of interest, individuals may aim not only to reduce their own experienced uncertainty but also to strategically increase others’ perceived uncertainty, e.g. via disinformation (*Machiavellian Intelligence Hypothesis*; [7]). Such deception can be valuable for deceivers if there is uncertainty about what others might do, but by deceiving others, individuals can manipulate them into a particular course of action that benefits themselves. In contrast, according to the *Vygotskian Intelligence Hypothesis* [51]*,* cognitive evolution (especially in humans) has been driven by uncertainty arising from the need for cooperative and communicative interactions to achieve joint goals. A similar position is reflected by yet another variant, the *Relationship Intelligence Hypothesis* [52]. This hypothesis aims to explain cognitive evolution in birds by arguing that long-term monogamous cooperative partnerships could be cognitively demanding given that partners may need to coordinate and keep track of each other’s actions [53]. A key assumption here is tracking the outcomes of previous interactions with partners will inform the optimal action for future interactions (see figure 2). However, in genetically monogamous relationships where the fitness of partners is tightly intertwined, individuals could also benefit from being maximally predictable towards each other [54]. This would reduce uncertainty and cognitive challenges (but see [55] for an argument about the role of interdependence among non-relatives in the evolution of human cooperation and cognition). To resolve these apparently contradictory perspectives, we suggest that it is vital to consider trade-offs across different socio-ecological challenges. Animals likely need to optimize solutions to different uncertainty problems that potentially require different behaviours, resulting in trade-offs. These trade-offs, such as needing to choose between different interaction partners across different contexts, could generate uncertainty and hence potentially select for cognitive abilities to navigate them. We turn to trade-offs and the role of cognition in overcoming them in the next section.

## 3. Socio-ecological trade-offs and the adaptive value of sociality and cognition

Animals encounter distinct sources of uncertainty across varying socio-ecological contexts and in interactions with different social partners. This results in trade-offs that could be resolved via cognition to obtain the benefits (and minimize the costs) of sociality. Crucially, individuals do not resolve social uncertainty in isolation, but as part of a social network of individuals interacting with and influencing each other. Thus, if individuals track information to reduce uncertainty about one socio-ecological context or social partner, this may limit their ability to invest in other contexts or partners owing to time and energy constraints. To identify such trade-offs and how animals resolve them, the idea of biological markets provides a useful framework [56]. A biological market entails interactions between members of a group that exchange the same or different behavioural commodities, such as grooming, food-sharing and alarm calling [56]. These commodities, and the information conveyed through their exchange, provide a currency to reduce uncertainty and maximize payoffs [57,58]. In some instances, this may require cognition, e.g. by tracking and remembering the outcomes of prior interactions to inform future decisions. For example, dwarf mongooses (*Helogale parvula*) keep track of their groupmates’ contributions to anti-predator vigilance, rewarding the most cooperative individuals with additional grooming [59]. However, although we can predict how animals might behave optimally to maximize payoffs in particular contexts, such as in social foraging [60], animals also seem to make ‘sub-optimal’ social decisions, such as associating with familiar partners that yield sub-optimal payoffs [61,62]. While associating with another individual could potentially yield higher rewards (e.g. greater energy intake during social foraging), associating with a known partner that presents less uncertainty may be better than incurring the costs of constantly acquiring additional information about an unfamiliar partner. Similarly, obtaining known rewards can sometimes be preferable compared with better but more variable rewards. This idea is often referred to as an exploration–exploitation trade-off [63]. The absence of ‘perfectly rational’ decision-making may in fact be driven by the constraints imposed by socio-ecological trade-offs: decisions are ecologically rather than psychologically rational [64]. Determining how such socio-ecological trade-offs occur and are resolved is central to understanding how sociality and cognition can coevolve. This is because market conditions and dynamics, such as the social structure of a group, determine the cognitive demands for individuals to make adaptive decisions. Conversely, through their decisions, individuals’ cognitive processes shape trade in the biological market. Below, we outline the cognitive implications of four important socio-ecological trade-offs for group-living animals.

The first, and most fundamental, trade-off arises between the benefits and costs of group living [65]. A classic example concerns balancing the benefits and costs of social foraging. The benefits of using social information to reduce uncertainty (e.g. about resource distributions or predation risk) are thought to be among the main drivers of group living [66,67], but these benefits trade off against the increased costs of competition [65]. The more animals depend on the benefits of sociality, the more salient information from and about the social environment can become [68]. If social information use provides a net benefit (e.g. by increasing foraging efficiency) then selection could act on underlying cognitive processes, such as attention to conspecifics [69] or social learning mechanisms [70]. For example, experiments show that highly social Mexican jays (*Aphelocoma wollweberi*) are more reliant on social information use than the less social California scrub jays (*Asclepias californica*) [71].

A second trade-off emerges if different socio-ecological challenges require different, potentially mutually exclusive behaviours. For instance, to obtain information about food and about foraging techniques, or to maintain close social relationships and provide social support, animals may need to stay in proximity to group members, but to avoid pathogens, they may need to distance themselves from others [29]. Animals might benefit from abilities to evaluate the current condition of others, for example using body condition as an indicator of foraging success or pathogen infection [72]. Given that the state of group members and environmental factors can change, individuals may also benefit from behavioural plasticity, for example by updating their knowledge about the foraging success or infection status of a conspecific [73]. Periods of uncertainty, such as when food is scarce or patchily distributed, may increase the value of learning from others [74]. To acquire social information, individuals could then be more likely to pay the costs of being infected with a pathogen.

Payoffs could also vary depending on whom an individual associates with, giving rise to a third trade-off between different interaction partners. This trade-off arises because deciding to interact with one individual reduces opportunities to interact with others. As the skills or knowledge states of potential social partners may vary across time, individuals may need to track information to decide with whom to associate and interact [22]. Trade-offs become acute if the value of a given social partner varies across contexts: for example, one individual might provide more reliable information about predators, but another might be more efficient at locating food. Furthermore, important trade-offs may arise in animals that form different ‘types’ of social relationships, such as long-term cooperative partnerships versus more transient, short-term acquaintances [62]. For example, in a recent field experiment, wild jackdaws were assigned to two treatment groups and rewarded for associating at automated feeders with individuals from their own treatment group. Individuals rapidly adjusted their foraging associations to maximize their payoffs, retaining associates that proved valuable and discarding those that did not [62]. Such flexible partner choice requires the ability to recognize different individuals, learn to associate others with outcomes (possibly by remembering the outcomes of past interactions with them) and, conceivably, to make inferences by observing and evaluating the interactions of others [75]. However, the experiment also revealed constraints on social flexibility, as jackdaws continued to associate with their pair-bonded mate irrespective of the foraging gains [62]. Therefore, where partners have highly interdependent fitness outcomes (as in monogamous birds), they may prioritize associating with each other over seeking better foraging opportunities (e.g. great tits (*Parus major*) [61]) or associating with other individuals that provide better short-term gains (e.g. jackdaws [62]).

Finally, once individuals have decided whom to interact with in a given context, they may need to overcome a fourth trade-off: deciding how exactly to behave in a social interaction. Taking one course of action entails the opportunity cost of pursuing another potentially suitable action, and therefore such decision-making can be conceived as a trade-off between the two. This may become more cognitively challenging if many different actions are available within the species’ behavioural repertoire, and if there is uncertainty as to which action would be most beneficial and which action to expect from a given social partner. For instance, individuals often use aggression to displace others from food sources. However, if other individuals acquire adaptive foraging information, it may sometimes pay dominants to refrain from aggression and instead show social tolerance to observe and learn new adaptive behaviour [76,77]. During a social interaction that can change dynamically, for example when social play changes from an affiliative interaction to an agonistic one, individuals could also benefit from flexibility to adjust their behaviour accordingly [78].

Flexibility in social behaviour to navigate these socio-ecological trade-offs may be underpinned by cognitive abilities, abilities that themselves can be tracked and responded to by conspecifics. For example, to decide with whom to form and maintain differentiated social relationships, individuals may have to invest in cognitive resources to evaluate, learn about and remember outcomes of social interactions with different social partners. If non-cognitive or simple heuristic-based solutions do not suffice, those (potentially energetically costly) cognitive resources could be ‘paid’ for by the net benefits that individuals can gain through those social relationships. Individuals can be expected to navigate these trade-offs mainly to maximize their own direct fitness but also the fitness of individuals with whom their interests are aligned, such as kin or mating partners in monogamous, interdependent partnerships. This has implications for how information is traded in the biological market, i.e. shared versus withheld. For example, the level of social tolerance, an indicator of shared interests in social relationships, can influence the extent to which social information, for example about food, can spread through groups [79]. Furthermore, information processing capacities may not only allow animals to navigate the biological market but may also themselves be ‘traded’ against other commodities. For example, knowledgeable foragers may trade their access to information for protection against predators by recruiting conspecifics [66]. Abilities for information-processing may also serve as a signal through which individuals can display their suitability as a social or mating partner [80]. When discussing how animals may benefit from cognitive abilities to navigate socio-ecological trade-offs, the constraints on cognitive abilities and their underlying neural architecture also need to be considered.

Overall, socio-ecological trade-offs present critical sources of uncertainty, generating selection for cognitive processes to facilitate adaptive social decisions. However, these trade-offs arise in social environments which are, to a degree, the outcomes of individual decisions. In the next section, we consider the processes through which decisions are made, and how these can exert bottom-up influences on social structure.

## 4. Perception–action feedback loops, individual decisions, and bottom-up processes

When overcoming socio-ecological trade-offs, animals are not merely passively exposed to social uncertainty; they can also influence the uncertainty they encounter through their own decisions and actions. For example, by avoiding a dominant individual, a subordinate can manage the associated uncertainty of being attacked or not. Two key ‘forms’ of uncertainty concern perception and action: perception uncertainty refers to aspects of environmental (e.g. social) stimuli, and action uncertainty relates to which action to pursue. To understand how uncertainty may be resolved, the concept of Bayesian updating provides a valuable tool by comparing *a priori* states (*prior*) to the likelihood of new evidence, to then update a set of predictions (*posterior*) [27,81]. This informational (Bayesian) approach is not specific to cognitive questions (c evolutionary biologists may consider genes as informative cues of environmental conditions [82]) and does not require specific assumptions about the mechanisms or implementation of cognition. Indeed, the precise implementation of this process will differ across individuals and species, for example in terms of their priors and their abilities to use new informational input for generating posteriors. In general terms, to minimize ‘prediction errors’ (i.e. mismatches between priors and new evidence), animals can use sensory input to update their predictions *and* they can act on and change their environment so that it matches their predictions. For example, an individual can collect social information about the (relative) dominance rank of another individual, or its value as a social foraging partner, which creates a prediction about future interactions with that individual. These predictions can also be enforced by specific action (which can precede sensory input), such as aggressive threat displays in the case of dominance interactions to ensure maintenance of the dominance hierarchy pre-emptively. These two processes, perception and action, can be viewed as two sides of a *perception–action feedback loop* [83,84] (figure 2) within which cognition is integrated (but see [85,86] for an alternative perspective), and that enable sensorimotor coordination in the socio-ecological environment [87,88].

Perception–action feedback loops in social decision-making can unfold via a range of physiological, cognitive and behavioural mechanisms. As we have mentioned, research often focuses on identifying ‘human-like’ abilities such as theory of mind [33], but often, individual discrimination, learning and memory can be sufficient to enable individuals to update their priors and make adaptive social decisions. In some instances, ‘simple’ social heuristics may suffice even without needing to learn and remember the outcome of past interactions [89]. For example, ‘emotional book-keeping’, whereby persistent emotional states towards a partner guide decisions, may enable animals to maintain and reap the benefits of social relationships with a reduced need to gather information and predict future interactions [90,91]. Similarly, cognitive biases can allow animals to reduce uncertainty and exploit opportunities without the need for more ‘costly’ information processing. For example, if individual quality tends to be consistent across contexts, knowledge about one trait of an individual, such as social status, can serve to attribute additional qualities to that individual, such as cooperativeness or knowledge (sometimes referred to as ‘halo effect’ [92]). Such a positive bias towards high-ranking individuals is seen in the social learning strategies of some animals, such as vervet monkeys (*Chlorocebus pygerhythrus*) [93]. Moreover, consistent inter-individual variation in behaviour (or ‘personality’) [94], such as sociability [95], can itself serve as a ‘signal’ as to how an individual is likely to behave, reducing social uncertainty and thus cognitive load. In contrast, social personalities may also select for and coevolve with information-processing abilities necessary for being responsive to individual variation [96,97].

Through the processes described above, individuals’ decisions influence the decisions of others, producing bottom-up consequences for local and global social structure and market dynamics [16]. For instance, if similar individuals choose to associate with each other (termed ‘homophily’ [98] or ‘phenotypic assortment’ [99]), this can generate phenotypically segregated social networks at the global level [99,100]. In biological markets, individuals may also choose to associate with others that differ from themselves, providing access to commodities they cannot access themselves [56,101]. They may also gain information by observing the interactions of third parties [75], such that individuals with good ‘reputations’ (e.g. as good foragers or valuable cooperators) become more central in the social network [21,22]. Crucially, the diverse cognitive mechanisms through which decisions are made have fundamental implications for overall network structure. If individuals strategically adjust their social associations by learning which partners currently provide the best rewards, they can effectively construct and optimize their own social niches in response to changing conditions [62,102]. Influential theoretical models suggest that such learned partner choice will re-wire social networks at a global level, producing clusters of ‘compatible’ individuals, which in turn favours the emergence and persistence of cooperation (e.g. [103,104]). However, such models do not account for the possibility that individuals may also seek to maintain valuable long-term relationships, which will constrain network plasticity. Thus, research into the emergence and evolutionary implications of global social structure must be grounded in an understanding of how individuals make decisions. We turn our attention to group-level properties, their top-down influence on individual decision-making and the resulting coevolutionary feedback, in the next section.

## 5. Group properties, top-down processes and the role of cognition for individual-to-society feedback loops

Group properties (e.g. size, composition, cohesion and hierarchies) are a consequence of inter-individual interactions [17,25,26]. Often, researchers seek to understand how these different group properties result in varying levels of ‘social complexity’ (e.g. [105]), but what matters for understanding individual-level cognition is what information individuals can perceive and act upon to maximize fitness [106]. To this end, uncertainty and the role of information should be considered across hierarchical levels of social structure, from individuals to dyads and groups [26]. An important question concerns to what extent animals may use information across different parts and levels of social structure. For example, uncertainty can be reduced via direct dyadic interactions and by eavesdropping on the dyadic interactions of others [75,107]. Furthermore, animals might rely on information from and about the emergent group-level structure, such as dominance hierarchies or the position of others in the social network [26]. If group-level properties provide the context for and are shaped by individual-level decisions, coevolutionary feedback between sociality and cognition can arise.

First, social relationships and group-level social (network) structure emerge from dyadic interactions [17], shaping individuals’ developmental trajectories and fitness. Social structure determines who an individual encounters throughout life, and the resulting exposure to social challenges and stimulation can shape cognitive development, as seen in fish [108], birds [47] and humans [109]. In some species, pre-existing social relationships (e.g. arising from dyadic interactions with parents) may also guide juveniles’ decisions as they explore, prune and consolidate other social interactions [110] (but see [111]). This top-down process, known as social inheritance [112], might reduce the cognitive challenges of navigating uncertainty in the social environment. For example, during social integration, juveniles may lack information to decide whom to associate with (i.e. uncertainty is high), but by inheriting their parents’ social associations, juveniles may manage such uncertainty (though this requires parental social associations to be relatively stable over time; see [113]). If social network structures are highly dynamic, such dynamism can also be a source of both uncertainty and valuable information [114], influencing selection on individual decision-making processes. For example, fission–fusion dynamics [115] can reduce the uncertainty induced by the trade-off between the need, on the one hand, to aggregate with others to acquire information and avoid predators and, on the other, to avoid competition and disease transmission [116]. However, the transient, changeable nature of social interactions and relationships in fission–fusion systems also generates uncertainty [117], posing cognitive challenges for individuals to track information and optimize their social position [118].

A second group-level phenomenon concerns the evolution of signalling systems. In animal societies where group members are in close proximity, this may favour the evolution of salient indicators of individuals’ affective states that act as signals, providing social information (e.g. about potential risks) that influences the behaviour of others [119]. This can even lead to the spread of affective states through groups through emotional contagion (or physiological state matching) [120], with group-level states then feeding back to influence the affective states of individuals. Other communicative signals (e.g. vocalizations or scent marks) do not require close proximity, but their perception and informative value still depends on social structure. The *Social Complexity Hypothesis* [121] (another variant of the Social Intelligence Hypothesis) suggests that social environments featuring greater uncertainty will select for greater diversity and flexibility in communicative signals. For instance, a greater number of potential social partners and social roles may select for larger vocal repertoires to enable more effective transmission of different types of information [121,122]. Similarly, societies featuring differentiated social relationships may favour the evolution of individually distinctive vocal signatures. This, in turn, will select for cognitive processes to integrate information about the identity of the caller with prior knowledge about the characteristics and reliability of that individual [123]. Thus, the transfer of information through communication determines the need for cognitive systems to process and act on the information, generating coevolutionary feedback between sociality and cognition.

Third, if individuals learn from each other, information can spread through social networks, giving rise to culture as a group-level phenomenon. Culture can create a tension between increasing and decreasing uncertainty, with subsequent impacts on how selection acts on cognition. On the one hand, learning from others reduces the need to invest in gathering and processing information, reducing the cognitive load on individuals. Copying the behaviour of the majority (‘conformist social learning’), for example provides a rapid shortcut to acquire information about local environmental conditions [124] and social norms [125,126]. Conversely, if environments change rapidly and there is substantial variation in individuals’ knowledge and skills, there may be strong selection for cognitive abilities to determine when to use social learning and who to learn from [127–129]. Indeed, there is evidence that in some animals, strategies for social information use can themselves be learned and updated through personal experience and by observing others [77,130], and in humans they may involve meta-cognition [131]. If reliance on cultural information increases, this will generate selection for more efficient means of social learning, enabling sequential improvements in cultural products, such as tools and technologies. As these products become ever more complex and difficult to learn alone, this cumulative culture may coevolve with specialized abilities to actively share information, such as language and teaching in humans [132]. The need to share information or withhold it from competitors is also likely to coevolve with abilities to use social information [133] and can shape patterns of social association. Thus, the emergence of culture both depends on and drives social structure [134].

The degree to which animals can exert bottom-up influence on group-level outcomes is likely to differ within and across species and contexts, influencing the coevolution of sociality and cognition. For example, in species with more diverse individual-level behavioural repertoires or behavioural flexibility, individuals might have more influence in shaping social structure via bottom-up processes. However, the groups that emerge from such bottom-up effects may intrinsically feature greater uncertainty. Thus, the group-level structures that emerge from individual interactions will in turn determine the degree to which selection favours individual variation, flexibility, and ability to exert influence.

## 6. Conclusions and future directions

The traditional perspective that social life is intrinsically ‘difficult’ and requires cognitive solutions is beginning to give rise to a view considering the coevolutionary interplay between sociality and cognition [16,20,21]. However, perhaps because of the nature of bidirectional causation, progress in understanding the role of social factors in cognitive evolution still lags behind that of external selection pressures. For instance, this special issue showcases the great progress that has been made in unravelling cognitive solutions to foraging problems, like trap-lining in butterflies and spatial memory for cached food in chickadees [135,136]. Research, including work on facial recognition in wasps [135] and cleaner–client mutualisms in fish [136], highlights the potential for similar progress in the social domain, but major questions remain. Below, we outline some outstanding questions and how they may be addressed in the future.

### (a) How does social life modulate uncertainty?

To understand the social sources of uncertainty that influence cognitive evolution, we must identify what decisions animals need to make and what information-processing abilities are required. Rather than grounding research in anthropocentric assumptions of what humans do, we advocate the use of tools, such as playbacks and automated experiments to manipulate the outcome of social interactions and the value of social partners [59,62,77,137]. By simulating changes in the dynamics of a biological market, researchers can then evaluate what information animals attend to and act upon to resolve social uncertainty.

### (b) What is the adaptive value of cognition in resolving social uncertainty?

Many correlational studies indicate that individuals that form and maintain strong social relationships gain fitness benefits [138]. However, the causal basis of these links, and whether they are underpinned by cognitive processes, remains unclear. Do individuals that are better socially integrated obtain such positions by means of their social cognition? If so, could individuals with socio-cognitive abilities that occupy central social positions also excel at other fitness-relevant tasks, such as foraging and predator avoidance, confounding the relationship between social integration and fitness? To address this, we must integrate research on the adaptive value of sociality and cognition. For instance, long-term monitoring of natural populations can help to reveal the degree to which social traits (e.g. network centrality or dominance) are shaped by information gained from experience throughout development. Moreover, as experimental studies of social decision-making (e.g. [62]) commonly reveal substantial variation in individual performance, there is ample scope for work linking this variation to its fitness consequences. Studies focusing on how animals resolve trade-offs (e.g. between long-term investment in social partners versus short-term gains of social plasticity) will provide important advances.

### (c) What are the bottom-up effects of individual decisions on emergent social structure?

To understand bottom-up effects, an important focus will be to address how (variation and plasticity in) individual decisions can give rise to social structure. By better understanding how individual decisions shape social structure, we will better understand how individuals’ actions affect both their and conspecifics’ exposure to uncertainty. Natural ecological events (e.g. extreme weather) [139], changes in group composition [114] and experimental manipulations [16] can also offer valuable opportunities to establish how decision-making affects the re-configuration of networks following disturbances. Here, advances in social network analysis will be essential, enabling explicit investigation of how individual actions drive dynamic changes in network structure [140].

### (d) How do (emergent) group-level properties arise and shape selection on individual decision-making?

Understanding the evolution of social traits (and the cognitive processes underpinning them) is complicated by the fact that individuals’ phenotypes depend not only on their own genotypes but also on those of their social partners. Recent advances in quantitative genetic modelling [141] now allow us to determine genetic variance in, and covariance among, individual cognitive and sociability phenotypes (e.g. dyadic bond strength and social network positions) and estimate direct and indirect contributions to fitness. In tandem with ongoing improvements in quantifying individual cognitive variation [142], such approaches will help us to understand how the structure of social networks influences the direction and strength of selection. Meanwhile, formal theoretical modelling will be invaluable to understand long-term evolutionary dynamics, as it forces us to be explicit in our assumptions and enables investigation of coevolutionary dynamics that are difficult to observe directly [143].

## Data Availability

This article has no additional data.
